# Developing a Third-Party Analytics Application Using Australia’s National Personal Health Records System: Case Study

**DOI:** 10.2196/medinform.7710

**Published:** 2018-04-24

**Authors:** Niranjan Bidargaddi, Yasmin van Kasteren, Peter Musiat, Michael Kidd

**Affiliations:** ^1^ Digital Psychiatry & Personal Health Informatics Lab College of Medicine and Public Health Flinders University Adelaide Australia; ^2^ South Australian Health and Medical Research Institute Adelaid Australia; ^3^ Flinders Digital Health Centre College of Nursing and Health Sciences Flinders University Adelaide Australia; ^4^ Department of Family & Community Medicine University of Toronto Toronto, ON Canada

**Keywords:** computer software applications, electronic health record, software design, medication compliance

## Abstract

**Background:**

My Health Record (MyHR) is Australia’s national electronic health record (EHR) system. Poor usability and functionality have resulted in low utility, affecting enrollment and participation rates by both patients and clinicians alike. Similar to apps on mobile phone app stores, innovative third-party applications of MyHR platform data can enhance the usefulness of the platform, but there is a paucity of research into the processes involved in developing third-party applications that integrate and use data from EHR systems.

**Objective:**

The research describes the challenges involved in pioneering the development of a patient and clinician Web-based software application for MyHR and insights resulting from this experience.

**Methods:**

This research uses a case study approach, investigating the development and implementation of *Actionable Intime Insights (AI*^2^*)*, a third-party application for MyHR, which translates Medicare claims records stored in MyHR into a clinically meaningful timeline visualization of health data for both patients and clinicians. This case study identifies the challenges encountered by the Personal Health Informatics team from Flinders University in the MyHR third-party application development environment.

**Results:**

The study presents a nuanced understanding of different data types and quality of data in MyHR and the complexities associated with developing secondary-use applications. Regulatory requirements associated with utilization of MyHR data, restrictions on visualizations of data, and processes of testing third-party applications were encountered during the development of the application.

**Conclusions:**

This study identified several processes, technical and regulatory barriers which, if addressed, can make MyHR a thriving ecosystem of health applications. It clearly identifies opportunities and considerations for the Australian Digital Health Agency and other national bodies wishing to encourage the development of new and innovative use cases for national EHRs.

## Introduction

### Background

Across the member countries of the Organisation for Economic Co-operation and Development, national electronic health records (EHRs) are at varying stages of implementation [1]. The overarching objective of national EHRs is to improve the delivery and administration of health care [1]. Australia is one of the countries that have implemented a national rollout of EHRs. The vision for My Health Record (MyHR), as with other national EHR systems, is to improve health care outcomes through coordinated care and the sharing of essential patient information at the point of care [2]. In the United Kingdom, which, like Australia, has a universal health care system, the National Health Service took a top-down approach in developing EHRs. In the United Kingdom, 97% (55 million) of health care consumers (hereafter referred to as patients) have online access to their Summary Care Record, which contains information on prescriptions, allergies, and adverse reactions and allows patients to access their record, book online appointments, and order repeat prescriptions. Only 0.4% of patients in the United Kingdom have ever used this service [3].

As in the United Kingdom, Australia’s MyHR is suffering from lack of buy-in from patients. Since its launch in 2012, uptake of MyHR has been insufficient to achieve the critical mass of patient participation necessary to galvanize health care provider engagement [4]. Unlike the United Kingdom, Australia adopted an opt-in system, and as of November 2017, only 22% of Australians were registered for MyHR. To mobilize health provider engagement, from 2018, MyHR will shift from an opt-in model to an opt-out model. Equally, health care providers have also been slow on the uptake as they have little incentive to use MyHR as a communication platform, because a vast majority already use practice management software with advanced functionalities including most of the information intended to be made available through MyHR [5]. For a national EHR system to succeed, both health care consumers and providers must buy into the system. However, it is not the uptake, but rather the usefulness of MyHR, which will ultimately drive the necessary widespread adoption and everyday use of MyHR for providers and patients alike [6]. Only then, the goals of improving health care outcomes with a national EHR system can be achieved.

Unlike the United Kingdom and Australia, the United States adopted a bottom-up approach to the development of a national EHR system. The strategy in the United States was a staged approach focusing on “meaningful use” [7]. The first stage saw the development of the necessary infrastructure for capturing and storing EHRs, and the second stage, currently in progress, incentivizes hospitals and health care providers to find ways to engage with electronic records in meaningful ways. The final stage will be the realization of the benefits of a national EHR system [7,8]. Research suggests that maximizing added value for patients is most likely to drive the widespread adoption of EHRs and realize the benefits and the outcomes of national EHR systems [6] because patient portals are important tools for engaging patients in their own health care [9-11].

In Australia, the initial focus of third-party software development for MyHR was on software integrating MyHR into existing clinical information systems. By clinical information systems, we mean computer-based systems for managing and storing in-patient or practice-based medical records and test results for the delivery of patient care in local settings such as a general practice (GP) or a hospital [12]. Since the inception of Australia’s national EHR, most conformant third-party applications have been clinical information systems or contracted service provider hosts [13]. In 2012-2013, 26 software vendors linked to MyHR through their clinical information systems, which included applications for GPs, hospitals, aged care providers, and pharmacies [14]. By 2016, a further 19 clinical information systems were registered plus 3 systems for contracted service providers to host or provide infrastructure for use by health care providers. On the other hand, development of software applications that are patient-centered has been limited.

In Australia, there are 2 factors that influence the usefulness of MyHR—the type of data sources available and the functionality of the system. First, MyHR is currently missing a coherent approach to automatically source data from health care providers and other repositories of health data, resulting in incomplete data. The most complete and up-to-date data currently available on MyHR are Medicare data. Medicare is Australia’s universal care provider. The Medicare claims database records medical intervention claims reimbursed through the Medical Benefits Scheme (MBS) and pharmaceutical prescriptions and dispensing claims reimbursed through the Prescription Benefit Scheme (PBS). Second, although access to fragmented medical history in a single place is one of the main perceived benefits and utility of EHRs [15], MyHR cannot currently create a collated health summary [5], which limits the usability of MyHR for the coordination of care.

In this research, we review the development of a third-party application called *Actionable Intime Insights (AI*^2^*)*, the first analytic software developed for MyHR by Digital Psychiatry & Personal Health Informatics Lab, Flinders University, Adelaide, South Australia. Designed as an application for both patients and clinicians, AI^2^ capitalizes on the demonstrated utility of Medicare claims data in understanding treatment [16,17] by collating and organizing medical visits and prescriptions and dispensing information from the Medicare database to allow patients and clinicians to view patient history in an intuitive timeline format. The research describes the challenges involved in pioneering the development of a patient and clinician Web-based software application for MyHR and insights from this experience. Recommendations for improvements to MyHR development environment are discussed.

### Data Stored in Health Record

**Figure 1:**
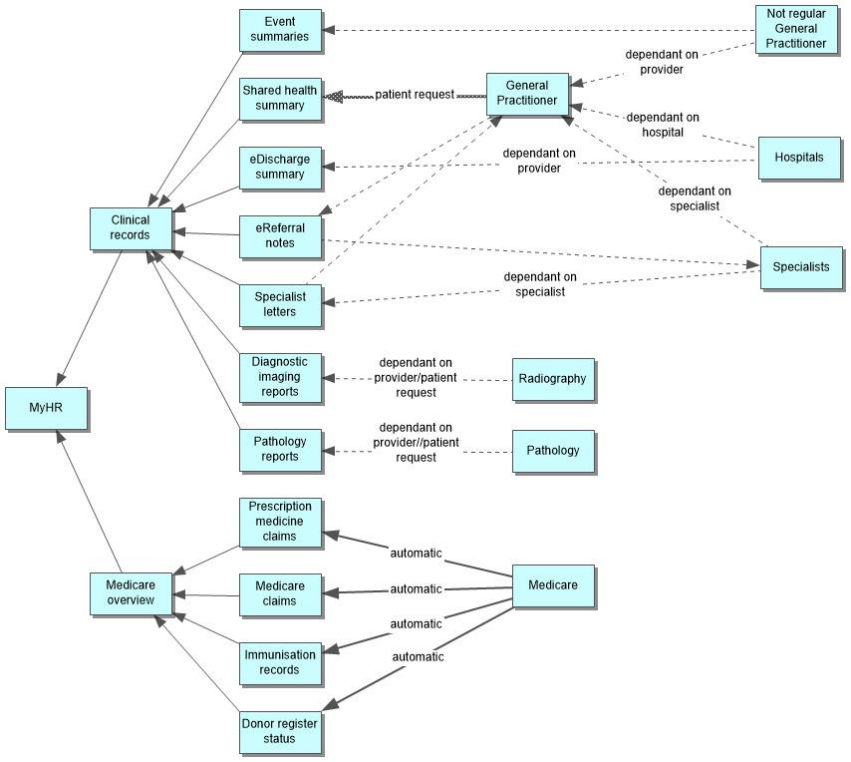
Overview of data input into My Health Record (myHR).

EHRs involve the exchange of information with other health care systems and repositories of health care data [18]. Developing a national EHR system is a complex challenge both in terms of design and implementation because of the involvement of multiple stakeholders and concerns over interoperability, privacy, and security [19]. MyHR collates data from many sources; however, different sources of data have different guidelines, procedures, and permissions that regulate their inclusion in MyHR. Figure 1 summarizes the sources of data that feed into MyHR and how they are generated as records in MyHR through the MyHR portal.

Health data stored in MyHR are programmatically accessible in a format known as views. There are 11 different views for different types of data as shown in Figure 1. Except for the Medicare view, all views are populated by data sourced from various clinical information systems used by different health care providers such as GPs, hospitals, or radiology and pathology clinics. The health data in each of these views are populated under different conditions with varying level of data completeness. First, data integration requires patients having a MyHR. Second, certain records, eg, GP records, require action by the GP and patients’ prior permission for them to be uploaded onto MyHR [20]. Third, health care providers must have the clinical information systems in place that can access and upload data to MyHR [21]. Finally, despite the availability of interfaces, not all available data are automatically extracted from these sources and populated into corresponding views. Data uploads depend on if health care systems and repositories are linked to MyHR and if they are set up to push data into MyHR automatically or on request. As a result of these factors, it has been claimed that health data in MyHR are often incomplete, thus making it unsuitable for patient care [22]. Incomplete data are also problematic for analysis.

A notable exception to the above is data stored under the Medicare view, which contains details directly populated from the Medicare claims database. It consists of a list of reimbursed MBS and PBS interventions. The Medicare records are complete and, on creation of a MyHR, the system automatically uploads retrospective data for 2 years from the date of activation of a MyHR account and then subsequently automatically updates records after each and every intervention.

### Medicare Data in My Health Record

Medicare data record reimbursement claims for dispensed prescriptions and administered interventions such as diagnostic and therapeutic procedures, oral and maxillofacial, diagnostic imaging, and pathology services, as well as data from allied health and optometry services.

**Table 1:** Data fields for Medicare data.

Medical Benefits Scheme data	Prescription Benefit Scheme data
Date of service	Generic name
Item number^a^	Brand
Item description^b^	Date prescribed
Name of practitioner	Date supplied
In hospital (yes/no)	Form and strength
	Quantity
	Number of repeats^c^
	Code

^a^Medicare Benefits Schedule outlining item numbers [23].

^b^For example, “consultation and consulting rooms, Level B” or “Initial Specialist Attendance, MRI scan for derangement of shoulder or its supporting structures.”

^c^Number of repeat prescriptions left.

**Figure 2:**
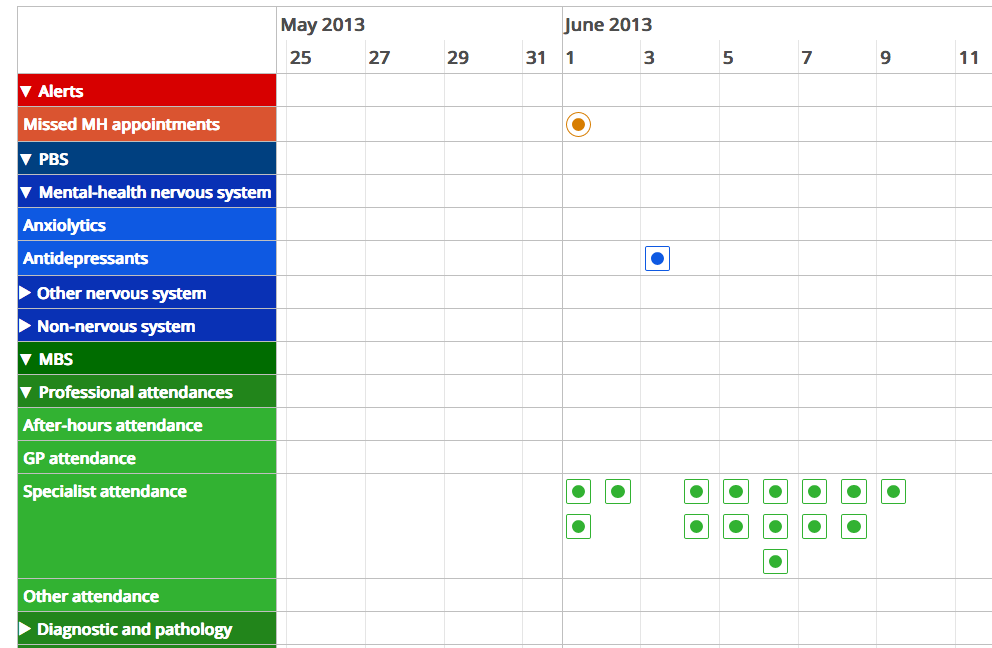
AI Squared Medical Benefits Scheme (MBS) and Prescription Benefit Scheme (PBS) data timeline visualization. GP: general practice.

The data detail the date of visit to a supplier of Medicare-funded services and the specific services or tests performed as described in the medical benefits schedule [23]. It also contains information about the type of medication, amount of medication supplied, and the date of supply. Table 1 shows the data fields as displayed in MyHR from Medicare’s PBS and the MBS. This information can currently be viewed by both patients and health care providers in the form of 2 separate tables.

### Benefits of AI Squared for Patients and Clinicians

AI^2^ is designed as a responsive Web application that visualizes MyHR Medicare view data in the form of a timeline view. It has patient and clinician interfaces but is designed to be a standalone application accessible by registered patients even when there is no interaction with health professionals involved. Thus, it is the first patient analytics-oriented third-party application interfacing with MyHR.

Medicare view data are a by-product of administrative claims processing. So, to provide an initial level of useful analytics, AI^2^ uses a simple taxonomy to map administratively used item types and prescription codes into clinically relevant categories. The clinical categories are based on the intervention provider type or medication names for each disorder. The mapping involved creating a parser that groups item numbers associated with similar service providers (eg, GPs, psychiatrists) and grouping of prescriptions associated with same disorders such as antidepressants. The result of grouping data and using the timeline format is that it is easier to quickly visualize a patients’ trajectory and gaps over time; see Figure 2.

The benefit for patients is that AI^2^ provides an intuitive timeline interface that can facilitate the management of their health, by collating pharmaceutical and health provider encounters. AI^2^ is most likely to benefit patients with severe mental illnesses or chronic disease, with ongoing medications and treatment through multiple care providers, by easing the burden of managing or recalling extensive history of polypharmacy and medical information across multiple health care providers [9,18,24].

For clinicians, understanding the patient history is an essential part of the patient interview [25]. For patients with complex conditions and in a time-constrained environment, a visual overview of the patient’s history assembled from the MBS and PBS data can improve accuracy, save time, and serve as a point of discussion between clinician and patient. The benefit of the timeline visualization of medication and medical services is that it can quickly reveal gaps in medications, and typical service uses patterns and changes, which provide clinical contexts relevant to assessment and treatments [26,27]. An overview of compliance with medication and treatment can also potentially reduce problems with polypharmacy and conflicts over care plans across multiple care providers [18,28,29].

## Methods

This research is a case study of a pioneering development of a third-party application for MyHR. Although the AI^2^ application has not been officially released as it is currently undergoing trials, it was nonetheless the first third-party application developed for MyHR. As such, it was a test case for the Australian Digital Health Agency and other developers of MyHR. This case study identifies the challenges encountered by the Personal Health Informatics team from Flinders University, in the development of a third-party application for MyHR. The research identifies opportunities and considerations for the Australian Digital Health Agency and other national bodies wishing to encourage the development of new use cases for national EHRs. Having a functional and efficient third-party application development environment is important because application can “stimulate open innovation and competition for products that deliver on consumer and health care provider expectations in the digital health space, and ultimately contribute to improved health care outcomes for people” [30].

## Results

### AI Squared Application

The development of the AI^2^ application involved implementing interface programs to access Medicare data, applying timeline visualizations on obtained Medicare data, and testing conformance requirements. The application was developed using open-source development and hosting tools. It is developed in Java Enterprise Edition using JBoss Seam and Hibernate frameworks. The database is created in PostgreSQL. JBoss Application servers and APACHE Web servers are required to run the compiled application. Implementation of a third-party application that interfaces with MyHR involved integration with 2 Web services known as Health care Identifiers verification (HI) Service, maintained by Medicare Australia, and MyHR Service from the Australian Digital Health Agency. HL7 SOAP protocol is used for integration with HI and MyHR Web services. The resulting application is developed to be registered as a stand-alone “health service” that can be accessed directly by the patients with or without a treating clinician and satisfy the requirements of 2012 Australian MyHR legislation. The source code of the application is available for the development of future patient-oriented applications and the corresponding author can be contacted to request access.

### Creating User Records in AI Squared Registration and Login Process

AI^2^ was designed with 2 types of users in mind: patients and clinicians. The application can be used independently either by patients, for self-monitoring, or by clinicians for checking on care plans or compliance or as a tool for both patient and clinician to verify patient history and discuss events, care plans, or treatments. Either the patient or the clinician can create an AI^2^ record.

Using test data, we created a patient record on MyHR. Creating a patient record on AI^2^ involves setting up a username, which is a mobile phone number and password as well as providing details required for the purposes of verifying Individual Health Identifier (IHI) with the HI service. To do this, patients can either provide their 16-digit IHI number, or alternatively provide other details (first name, last name, date of birth, gender, and Medicare number). The application sends this information to HI service for verification, and on receiving a valid user confirmation, a user record is created in the AI^2^ database.

The registration page for clinicians is similar, as it also involves entering a mobile number and password similar to the patient users. The registration page also has the capacity to collect the 16-digit Healthcare Provider Identifier (HPI-I), another unique identifier used by MyHR for verifying health professionals. All clinicians automatically have a Healthcare Provider Number, which is their Australian Health Practitioner Regulation Agency ID number with the addition of “800361” digits before [31]. The application sends this information to HI service’s “Healthcare provider directory search*”* application programming interfaces (API) for verification purposes.

### Process of Accessing Data From My Health Record

The process for fetching data from MyHR within AI^2^ involves, first, verifying that the individual has an activated MyHR, and on successful confirmation, gaining access to MyHR database. After receiving successful confirmation, the application then fetches health data using “Getview” APIs. The application is programmed to extract the latest records from MyHR whenever a patient profile is opened by either a patient or a clinician. Patients can also view a list of AI^2^-registered clinicians and can control which of these clinicians has access to their data using a connect or disconnect button.

Registered clinicians have to be connected to a patient user before they can view the patient’s record. The application can be configured to allow clinicians either to be automatically connected to all or selectively connected to registered patient users. If a clinician is not designated to have automatic connection with all individuals, they can search for patients by name, date of birth, gender, and Medicare number to find matching patients and connect, as is current practice in hospitals.

### Applying Timeline Visualizations on My Health Record Data

**Figure 3:**
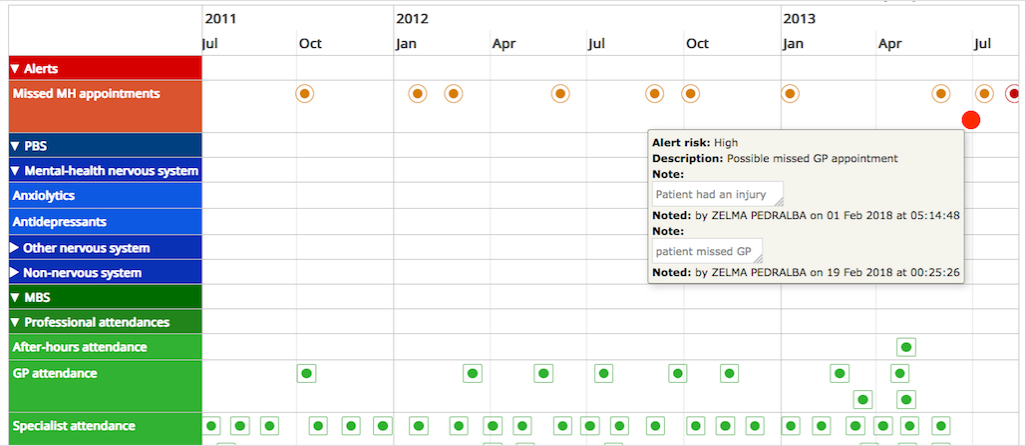
Timeline visualization of Medical Benefits Scheme (MBS) data over a number of years. PBS: Prescription Benefit Scheme.

The AI^2^ application applies a visually rich interactive timeline visualization on Medicare view data, implemented using vis.js and timeline.js, 2 open-source, browser-based visualization libraries. The timeline visualization displays each MBS and PBS claim stored in Medicare view as an event against a timescale on horizontal axis (see Figures 2 and 3). The events are shown at the corresponding start date position displayed on the horizontal axis, and the width of the event box can be adjusted to the corresponding end date position if it exists. Each event is associated with a group, and all events with the same group are shown in the same row or adjacent rows as a box, and the common value of their group property is used as a label at the left edge of the timeline. A simple taxonomy was developed to create a hierarchical grouping of PBS prescriptions and MBS interventions such that they are clinically meaningful and that variables required for assigning them into the groups were available in the Medicare view data. PBS prescriptions were grouped into generic medication classes, and MBS interventions were grouped into 4 categories—namely, GPs, specialists, imaging, and pathology. Within each MBS group, there were 2 subgroups based on if the service was provided in hospital or out of hospital, and within each subgroup, further levels were based on the nature of service. The group labels are displayed at the left side of the timeline visualization. Each MBS intervention and PBS prescription contained in the Medicare view (also see Figure 2) is then displayed as an event on timeline in the row of the group it belongs to. The timescale on the horizontal axis can be adjusted with zooming options, which dynamically scale the visualized data. For each event box, additional contextual information is displayed either in the box or on the top of a banner. In the case of MBS interventions, contextual information includes the description of the claim such as “Optometrist visit” and the details of the service provider as well as if the service was provided “in hospital” or “out of hospital.” For PBS prescriptions, the contextual information includes prescription name, dispensed date, and number of medications supplied. In addition to the above visualization, a simple notes entry page was also included. This was designed to encourage patients and health professionals to record insights or alternatively enable patients to make notes or observations they may like to follow up on.

### Process of Developing Software for My Health Record

MyHR consists of a centralized infrastructure to verify, obtain, and transfer health information of individuals from repositories holding health and clinical documents. It is designed to interact with several distributed repositories, some of which are established and managed centrally by the Australian Digital Health Agency, and others are maintained by registered external organizations, including Medicare. The central infrastructure is designed to query and identify the repositories that contain information about an individual patient using their unique identifier, known as Individual Healthcare Identifier (IHI) and to collate available data on request from the repositories.

**Figure 4:**
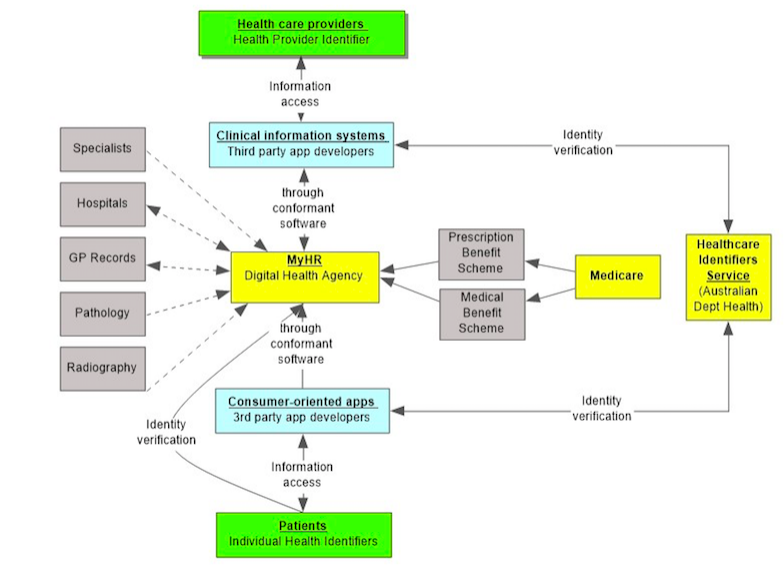
Links between systems in the third-party application development environment. Continuous lines represent automatic push of information or data. Dashed lines represent push data on request of information or data. GP: general practice; MyHR: My Health Record.

Figure 4 illustrates the way in which repositories and providers connect via MyHR.

### Regulatory Requirements for Using My Health Record

#### Registering as an Application Developer

Developing and administering the AI^2^ application involves hosting the infrastructure on a server and meeting eligibility criteria to be a host organization by the Australian Digital Health Agency. The host organization, in this case, Flinders University, must adopt a MyHR use policy, register to be a MyHR “participating organization,” and apply to obtain a Health Provider Identifier. The purpose of MyHR’s use policy was to ensure that organizations are accessing data through conformant software for providing health care only.

To develop a MyHR application for patients and or clinicians, the first step was to register as an application developer with Medicare and the Australian Digital Health Agency , after which access is granted to a development environment and a test kit containing sample data for testing and descriptions of supported integration use cases.

The Personal Health Informatics team from Flinders University was registered as a software developer with Medicare Australia and the Australian Digital Health Agency to gain access to developer and testing environments. At the time of the development of AI^2^, access was restricted to applications developed for the purposes of providing “health service” as defined in the Privacy Act 1988 (Cwlth) as follows:

(a) an activity performed in relation to an individual that is intended or claimed (expressly or otherwise) by the individual or the person performing it:

(i) to assess, record, maintain or improve the individual’s health; or

(ii) to diagnose the individual’s illness or disability; or

(iii) to treat the individual’s illness or disability or suspected illness or disability; or

(b) the dispensing on prescription of a drug or medicinal preparation by a pharmacist.

The interpretation of this act presupposes the involvement of a clinician. There were no special provisions for the development of patient portals, which therefore were also required to meet the definition of providing a “health service.” Thus, a case was argued with the Department of Health that the analysis provided by the AI^2^ met the criteria of providing a “health service” by providing patients with health data insights. Flinders University was given as a unique 16-digit identifier as a “health services provider” under the auspices of Healthcare Identifiers Act 2010 (Cwlth) (21).

**Figure 5:**
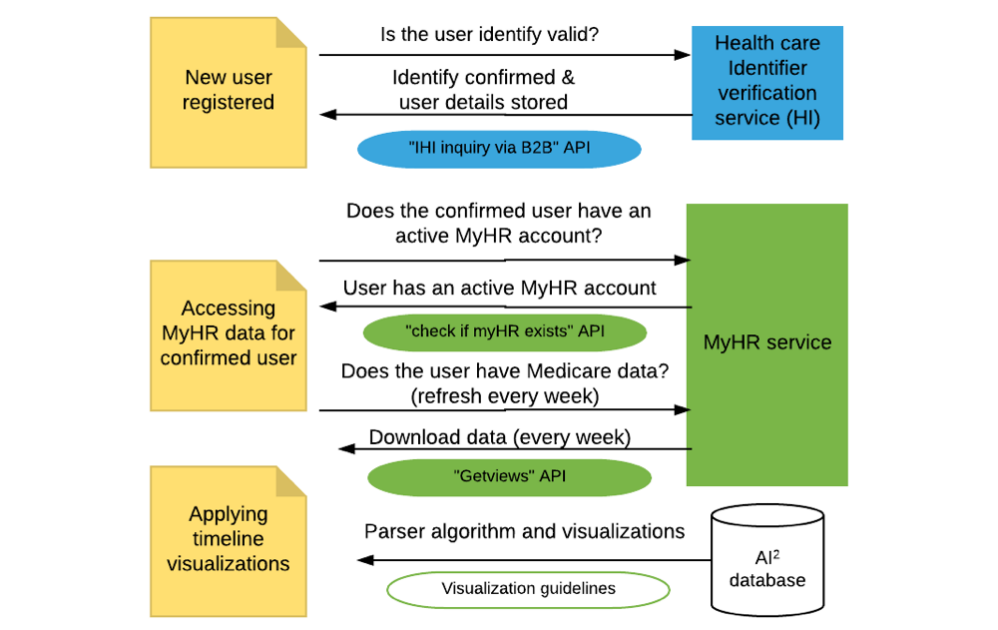
Software integration with Healthcare Identifiers verification service (HI) and My Health Record (MyHR) service. AI^2^: AI Squared; B2B API: Business 2 Business application programming interfaces.

#### Software Implementation

Figure 5 shows the different software components a third part party application must implement. AI^2^ had to integrate the Business 2 Business API’s functionality from the HI service into the application to verify the identity of their application users via the HI service to exchange data with MyHR by integrating. Third-party applications that can successfully verify users through the HI service are then eligible to read and write MyHR data using API functionality offered by MyHR.

#### Technical Conformance Testing and Gaining Access to the My Health Record Production Server

As would be expected on a national EHR, there are strict technical and intended use requirements. AI^2^ and other third-party software that integrates with MyHR had to meet conformance requirements of both the HI service and the My Health Record to demonstrate that the application could first exchange information with the HI service, and second, pass conformance tests to ensure the information exchange was consistent with approved use cases and rendering guidelines for displaying information from MyHR. Each test, 4 tests in all, took between 3 and 6 months.

The testing process involved first creating test cases, and subsequently, remotely assessing these test cases using the sample data provided in the test kit. Sample datasets in the test kit were mainly aimed at testing the authentication and data access process and not utilization needs. They neither contained longitudinal records nor did they have a sufficient number of test cases required for refining the visualization categories. Thus, it was necessary for us to use data sourced from a different study to develop the timeline analytics [32].

The first test, with the HI Service, the Notice of Connection (NOC) test, involved taking a screenshot and log file based on test cases, which were then approved by testers at the Department of Health.

After completing the NOC test, the second test, the HI conformance test, was conducted by IV&V Australia, an accredited tester at a cost of Aus $10,000. On passing the HI service conformance tests, the Department of Health issued an approval letter with details to gain access to the production HI Service.

The third test, the MyHR NOC test, was carried out by Ventura Inc. at no cost. Testing involved verifying that appropriate warnings and alerts were displayed in the application for incorrect individual details included in sample data. A tester from Accenture PLC remotely monitored the application, while the developer tested different patient and clinician scenarios, also at no cost. The fourth and final test, the MyHR conformance test of rendering guidelines, was done via a self-assessment form.

Displaying timeline visualizations of Medicare data obtained from MyHR required overcoming several standards-related challenges. To meet the software conformance standards, health data had to be displayed in a predefined format and style guidelines set by the Australian Digital Health Agency [33]. The guideline specifies 67 different requirements covering font size, format, and structure, and not all of which were applicable to the formats and style of timeline visualizations. As a workaround, we created 2 different data visualizations on the same Medicare data, the first using rendering in the approved style as prescribed by the Australian Digital Health Agency and the second visualization using the desired timeline visualizations described above. Subject to implementation of a conformant approach, no restrictions applied to alternative secondary visualizations.

On completion of all 4 conformance tests, the Department of Health issued a letter of approval, granting access to the MyHR production server through the AI^2^ application.

#### Requirements for Organizations to Use the AI Squared Application

To use the AI^2^ application, an organization must register with the Australian Digital Health Agency and obtain an HPI-O number, which is a unique 16-digit organization number verifiable by the HI service as well as a National Authentication Service for Health Public Key Infrastructure Certificate, a digital certificate for activating the conformant software AI^2^ application. Both these details need to be keyed into AI^2^ before the platform can make connections with live MyHR. Both clinicians and patients have to read and consent to the terms of use for the AI^2^ service and the privacy policy of the AI^2^ application, which were developed in consultation with the Flinders University legal team. The privacy policy contains information on user obligations and outlines how information sourced from MyHR are used and managed. After this process, the South Australian Health and Medical Research Institute is registered as the “participating organization,” and an instance of the AI^2^ application for supporting psychiatric patients is hosted on Nectar, a cloud server infrastructure available for Australian university researchers under the AI^2^ website [34].

## Discussion

### Overview

Developing a third-party patient application for EHRs has been previously attempted and deemed difficult [35]. This paper documents the process and challenges encountered in the development of an innovative third-party patient and clinician application, the AI^2^ application, which displays Medicare data from MyHR in an intuitive timeline format. Although there were numerous third-party clinical information applications being developed for MyHR, AI^2^ was the first application also designed for patients. Results identified several challenges encountered in the process of developing the application that can be grouped in 4 categories: (1) regulations related to use of MyHR in applications, (2) type of applications of MyHR data, (3) issues related to data processing, and (4) regulations related to testing.

### Regulations Related to Use of My Health Record Data in Applications

First, at the time of the development of AI^2^, the focus of development of third-party applications was predominantly on development of clinical health information systems. A major challenge in the development of AI^2^ was to understand how a patient-centered third-party application might meet these requirements.

All third-party conformant software has to interface with MyHR via the provider portal. To do so, at the time, providers had to be recognized as a “health service provider,” defined by the MyHR legislation [36]. The legislation stipulates that eligible entities can only utilize MyHR data for the purpose of providing “health services” as defined in the Privacy Act 1988 (Cwlth), regulation designed with development of clinical information systems in mind. Several revisions were made to act in 2006 after consultation and review, but the definition of use has not changed. This definition is problematic for the development of patient-focused third-party applications, in particular, for the new category of applications widely known as online or Internet personal health interventions, standalone applications of personal health interventions which have been shown to be effective in health, eg, in treatment of depression [37] or cardiovascular rehabilitation [38].

Circumventing the definition of “health service provider” has 2 possible interpretations, one is to consider the application itself as a “health service provider”, and the other is to consider the organizations creating and hosting standalone patient-oriented applications of MyHR as “health service” providers. Either way, defining the software or the software development companies as “health service” may raise issue of regulation.

Treating the software itself as a “health service provider” feeds into the growing debate on the need for standards and regulation-related health service provision for standalone applications of personal health interventions [39,40]. In Australia, software offering personal health interventions might then arguably fall under the auspices of the Therapeutic Goods Administration, which regulates goods used in “preventing, diagnosing, curing or alleviating a disease, ailment, defect or injury in persons” or for “influencing, inhibiting or modifying a physiological process in persons,” [41] and similar legislation would apply in other countries.

Treating software development companies as health service providers may result in more opportunities for creating new applications using MyHR data; however, health services providers, in particular, professional health services providers, are strictly regulated [42]. Defining software development companies as “health service providers” may potentially require registration and or accreditation, and it might also change the way in which complaints are handled [42].

Another challenge raised by framing MyHR third-party software development as “health service” provision is a restriction on use and, in particular, restrictions on use for research and analysis which do not meet the criteria of the My Health Record 2012 (Cwlth) legislation. Paradoxically, the restriction on use only applies to retrieving data from MyHR. Once the information is lawfully obtained from MyHR, local terms of use and privacy policy within the application can be applied for subsequent utilization of downloaded data to support new use cases such as in the case of AI^2^, future plans for providing treatment decision support, data linkage endeavors, or recruitment for clinical trials [43]. Although recently, the Australian Digital Health Agency has begun investigating frameworks for the secondary use of data in the MyHR system for research, policy planning, system use, quality improvement, and evaluation activities.

### My Health Record Data Application Use Case Considerations—Completeness of Records

The quality of and completeness of records/data available via MyHR varies substantially and is a major limitation for the development of new use cases with MyHR. The completeness and therefore the value of information under each of the views depends on, for some records such as GP-shared summaries, whether the patient permission to upload has been granted, whether the service provider has the technology to upload to MyHR, and whether the data upload happens automatically. Currently, only Medicare data are complete and up-to-date and, therefore, potentially useful for innovative analytics and clinical trial use cases.

### Data Processing Challenges for Analytics

Developing and using MyHR data for analytics applications for MyHR also presents 2 data processing challenges. The first relates to standards and interoperability. The data held by MyHR from the clinical repositories are analogous to shared file repositories, such as Dropbox. With the exception of Medicare data, data in MyHR from sources such as hospital and GPs are a collection of clinical documents with free text information. These data are characterized by clinical terminologies and jargons specific to the repositories in which they were created. To use this kind of data for analytics requires understanding and developing approaches to translate these terminologies using natural language processing analysis. Equally devising taxonomies, particularly suitable for analytics, can help reduce data complexity and potentially accelerate development of decision support applications using the data from MyHR. Efforts to reduce complexity in data using taxonomies have been attempted in apps and wearable devices [44] and online marketing [45].

The second challenge relates to visualization and rendering of data sourced from MyHR. Results showed that a time-consuming work around was needed to meet the very prescriptive rendering guidelines needed to obtain conformance, even though the purpose of the application was to develop an alternative visualization of data. AI^2^ overcame this challenge by implementing both the default rendering and the new timeline visualization; however, developing the work around was time-consuming and ultimately did not contribute to the usefulness of the application. Relaxing the rendering guidelines would allow software developers to create new visualizations of data.

### Regulations Related to Development and Testing

The ease of third-party application integration with MyHR system is another important factor in the development of secondary applications. Development with MyHR involves integration with 2 separate but interrelated services, which are coordinated by 2 different organizations. Medicare is responsible for the integration of user identity verification, and the Australian Digital Health Agency is responsible for the integration of provider identity verification. This dual verification process creates a complex workflow for application developers, particularly for startups and small businesses with limited resources. In addition, new applications have to be tested separately for an active connection and conformance to standards for each of these services. This complex process not only involves testing several elaborate scenarios, which may or may not be within the scope of the developed application, but also involves conducting these tests with 4 different teams. In this project, the time and resource allocated for meeting conformance requirements were substantially higher than was justified by the actual use cases. Although significant support is offered by the team at the Australian Digital Health Agency to help developers navigate the process, streamlining the testing process could actually reduce the time and costs for both application developers and the agency.

Streamlining integration and testing processes within MyHR will reduce the costs of third-party application development and reduce the time to market. It is more likely to create the impetus for an ecosystem of applications with innovative use cases of health data to emerge as it has for in the consumer wearables and health application spaces. Improvements to the way in which software developers can integrate and work with MyHR will ultimately both improve engagement with and generate value for MyHR. Finally, improving the size and richness of test data provided to application developers such that it reflects complete records of several patients can also assist in helping development of new applications.

### Conclusions

Given the substantial investment by the Australian Government, and other governments worldwide, in developing National EHR system, MyHR system operators and other national EHR authorities are increasingly recognizing the value of innovative third-party applications in adding value for consumers by better engaging consumers in their own health care and for clinicians, through the addition of analytics to clinical portals. Innovative applications have a key role in realizing the initial vision for MyHR of improving health care outcomes and gaining efficiencies in the delivery of health care services. Consequently, it is important that the development environment facilitates rather than hinders third-party application development to attract and capitalize on research and entrepreneurial activities in this space.

## Abbreviations

AI ^2^: Actionable Intime Insights application
API: application programming interface
Cwlth: Commonwealth
EHR: electronic health record
HI: Healthcare Identifier verification service
HPI: Healthcare Provider Identifier
IHI: Individual Health Identifier
MBS: Medical Benefits Scheme
MyHR: My Health Record
NOC: Notice of Connection
PBS: Prescription Benefit Scheme
